# The Nervous System, Anatomical and Physiological: In Which the Functions of the Various Parts of the Brain Are for the First Time Assigned; and to Which Is Prefixed, Some Account of the Author's Earliest Discoveries, of Which the More Recent Doctrine of Bell, Magendie, &c. Is Shown to Be at Once a Plagiarism, an Inversion, and a Blunder, Associated with Useless Experiments, Which They Have Neither Understood nor Explained. Being the First Volume of an Original System of Physiology, Adapted to the Advanced State of Anatomy

**Published:** 1835-04-01

**Authors:** 


*The Nervous System, Anatomical and Physiological: in which the Functions of the various Parts of the Brain are for the first Time assigned; and to which is prefixed, some Account of the Author's earliest Discoveries, of which the more recent Doctrine of Bell, Magendie, &c. is shown to be at once a Plagiarism, an Inversion, and a Blunder, associated with useless Experiments, which they have neither understood nor explained. Being the First Volume of an original System of Physiology, adapted to the advanced State of Anatomy.* By Alexander Walker, Author of "Physiognomy founded on Physiology."—London, 1834. 8vo. pp. 704.

We looked through Mr. Walker's book with considerable interest. The physiological world has been of late years so much divided, that we hailed with pleasure the announcement of discoveries earlier than those which have formed the frequent subject of contest, and hoped that the existing feuds would be put an end to, by the appearance of a claimant of earlier date: but, although we have found in Mr. Walker's work the evidence of earlier publication than Bell, and Magendie, and Mayo, we are disappointed, on the other hand, to find that Mr. Walker's claims are not of that specific nature which will allow us to own that he has anticipated the physiologists in question. With a strong turn for metaphysico-physiological speculation, considerable originality, and often with a critical justness which we have admired, Mr. Walker seems rather to have speculated *beyond* his own time,—and certainly praiseworthily *for* his time,—than to have actually anticipated what others have since accomplished.

We hardly, indeed, know how to recommend, how to condemn, how to praise this work; we cannot wholly condemn it, there is so much ingenious writing and clever anticipation in it; we cannot wholly praise and recommend it, for the errors incorporated in it are so many, that they would greatly mislead a student, who should attempt to learn from it the physiology of the nervous system. The work, however, is probably intended for masters, not for students; and the ability displayed in it undoubtedly makes it worthy of their perusal.

Mr. Walker is a very clever man,—not for all time, but for a portion of a century: if his writings had become generally known when first produced, they would have won him laurels which would have lasted a life-time: produced at present, they are like a rich dress, not worn, of fifty years ago, which suits not the present mode; for the fashion of a past theory, like the dress of the Chevalier Grammont, (if it could now reappear,) which was lost in a quicksand, is a thing to be admired, not used.

It is clearly shewn, by Mr. Walker, that Galen and Willis (no mean name,) had conceived or adopted the idea that different nerves have different offices, which are determined by, or correspond with, the difference of their origins. Mr. Walker, following in this track, supposes the anterior branches of the spinal nerves to be sentient nerves; which, by the anterior fasciculi of the spinal marrow, connect themselves with the brain, which decompounds and reunites the impressions they received, and originates reflection, appetites, desires; that from the cerebrum the half-digested thoughts are transmitted by the upper peduncles of the cerebellum to that organ; that there being further concocted, they excite impulses, which go down the posterior parts of the spinal marrow to the posterior roots of the nerves, to issue the mandates of the will.

This is all very specious; but what are the facts, as now demonstrated? The discoveries of Magendie, Bell, and Mayo, show the reverse of Mr. Walker's theory to be true: they prove that the anterior roots of the spinal nerves, and their equivalents in the fifth, are for motion; the posterior for sensation. Mr. Walker says, that the authors we have named have taken his thoughts, and inverted them: this would be good Old-Bailey evidence, in the case of a coat or pair of trowsers, but surely not in an equity suit touching the property of ideas. If Titius takes the nether garment of Caius, and inverts it, we allow that it still remains the property of Caius; but, if B claims the reverse of what A vindicates as his own, we cannot concede that B is a plagiary. Our author, however, will have it, that the eminent physiologists with whom he is wroth have stolen their theories entirely from him, and have nothing of their own but their blunders; or, as the epigrammatist says,

> " Jack stole his discourse from the fam'd Dr. Brown,
> But, by reading it damnably, made it his own."

We will conclude our notice of Mr. Walker's book with the following extract, which will give an idea of the vein in which it is written, and of the author's controversial style.

*" Inversion of the Writer's Discovery by Sir C. Bell, in* 1821 *or* 1822.*

" Sir C. Bell afterwards abandoned this doctrine, and precisely inverted the doctrine of the present writer; making the *anterior* nerves those of *volition*, and the *posterior* those of *sensation.*

" It is remarkable, however, that, among other writers, returning more or less to the doctrine of the present writer, which has never varied, Bellingeri asserts that the roots which take their origin in the *posterior columns* do convey impressions that excite *muscular contraction*, and govern muscles of extension! while Schoepf asserts that the *posterior* columns and nerves, as well as the anterior, are subservient to *voluntary motion!!* and, finally, Magendie asserts that, when the *anterior roots* are irritated, appearances of *sensibility* are caused, and, when the *posterior roots* are irritated, *muscular contractions* are in some measure caused!!! —The error which these statements involve shall afterwards be made evident: their approximation to those of the writer is the object of the present notice.

" The writer has already stated, that whatever merit belongs to his observations may be classed under one or other of the following heads: 1st, priority in the ascription of distinct functions to equally distinct parts; and, 2dly, the precise accuracy of that ascription.

*" Distance of Time even between the Writer's last Repetition of his Doctrine, and its Inversion by Sir C. Bell and others.*

" Now, as to the first even of these heads, it is still more remarkable than all the author has yet stated, that, long before Sir C. Bell had followed the writer even in ascribing distinct functions —sensation and volition, to distinct columns and nerves, (he appears first to have done so in his Papers to the Royal Society in 1821 and 1822, or rather in the Exposition prefixed to them in 1824,) the present writer had repeated his previous doctrines in several Numbers of Dr. Thomson's ' Annals of Philosophy' for 1815, in consequence of a dispute on the subject with the late Dr. Leach, of the British Museum. Thus it was not till at least six years after this last repetition of the present writer's doctrine, that Sir C. Bell ventured to follow him, even in ascribing distinct functions, sensation and volition, to distinct columns and nerves!

" As to Magendie, his experiments were not published till July or August, 1822; and, as they were published only in the form of a very short notice, it is presumed that they had been but recently

* " On again glancing at Sir C. Bell's Papers to the Royal Society in 1821 and 1822, it appears that the writer should not give him credit for this inversion, till the publication of his 'Exposition,' prefixed to their republication, in 1824.—It may perhaps be said to have been *indicated* in the Papers of 1821 and 1822, so that it was not difficult for any one to make the additional step."

performed. In 1822 or 1823, Magendie thought he found that no pain was caused by irritating the surface of the spinal cord between the anterior roots of the nerves of each side, but that the muscles supplied by them were thrown into violent contraction; and, on the contrary, that acute pain was caused by irritating the posterior columns of the spine between the posterior roots of the nerves. He does not, however, then say whether or not, in the latter case, he found any contraction to be caused in the muscles supplied by these nerves; and he has since, at least, modified these views.

"As to Magendie, a few additional words are required. He would appear to have cleverly anticipated Sir C. Bell just before he had concluded, as he would speedily, that sensation and volition belonged to different columns. Even now he seems to contemplate further operations of the same kind: he tells us that he has been 'engaged at intervals in experiments on the use of the different parts of the brain, and will make known the results as soon as they appear worthy of public notice.' It was under a similar provision ('nous nous occupons depuis quelque temps d'expériences directes sur ce point,' &c.) that he thought he was snatching something good from Sir C. Bell, when he adroitly appropriated and improved his remarks on the spinal nerves, not knowing the precise history of these discoveries. But both M. Magendie and Sir C. Bell must learn that printed dates, where actual publication is proved by ample living evidence, will always constitute the sole test of priority. In this, as in other things, the French too often act *en corsaire.*

"Of Mr. Mayo the writer wishes to speak with respect. His conduct in discussion with Sir C. Bell, his elaborate work on Physiology, and his invaluable translation of Reil's papers on the Brain, are titles to the respect of every physiologist. Mr. Mayo mentions, in his Anatomical and Physiological Commentaries, that, being aware of the composition of the trigeminal nerve at its origin being the same as that of the spinal nerves, and having ascertained that its twigs, which pass through the ganglion of Gasserius, are destined for sensation only, while those which do not are intended for exciting muscular motion, he was led to conjecture that the posterior and anterior roots of the spinal nerves were similarly circumstanced; and he adds, that he was engaged in experiments to determine the fact, when the publication of Magendie's rendered his unnecessary.* (P. 70.)

* "Anat. and Physiol. Comment., No. ii., pp. 9 and 10."

